# Pan-Cancer Genome-Wide DNA Methylation Analyses Revealed That Hypermethylation Influences 3D Architecture and Gene Expression Dysregulation in HOXA Locus During Carcinogenesis of Cancers

**DOI:** 10.3389/fcell.2021.649168

**Published:** 2021-03-18

**Authors:** Gang Liu, Zhenhao Liu, Xiaomeng Sun, Xiaoqiong Xia, Yunhe Liu, Lei Liu

**Affiliations:** ^1^Institute of Biomedical Sciences, Fudan University, Shanghai, China; ^2^Key Laboratory of Carcinogenesis and Cancer Invasion, Ministry of Education, Key Laboratory of Carcinogenesis, National Health and Family Planning Commission, Xiangya Hospital, Central South University, Changsha, China; ^3^Shanghai Center for Bioinformation Technology, Shanghai, China

**Keywords:** pan-cancer, DNA methylation, biomarker, Hi-C, hox, carcinogenesis

## Abstract

DNA methylation dysregulation during carcinogenesis has been widely discussed in recent years. However, the pan-cancer DNA methylation biomarkers and corresponding biological mechanisms were seldom investigated. We identified differentially methylated sites and regions from 5,056 The Cancer Genome Atlas (TCGA) samples across 10 cancer types and then validated the findings using 48 manually annotated datasets consisting of 3,394 samples across nine cancer types from Gene Expression Omnibus (GEO). All samples’ DNA methylation profile was evaluated with Illumina 450K microarray to narrow down the batch effect. Nine regions were identified as commonly differentially methylated regions across cancers in TCGA and GEO cohorts. Among these regions, a DNA fragment consisting of ∼1,400 bp detected inside the HOXA locus instead of the boundary may relate to the co-expression attenuation of genes inside the locus during carcinogenesis. We further analyzed the 3D DNA interaction profile by the publicly accessible Hi-C database. Consistently, the HOXA locus in normal cell lines compromised isolated topological domains while merging to the domain nearby in cancer cell lines. In conclusion, the dysregulation of the HOXA locus provides a novel insight into pan-cancer carcinogenesis.

## Introduction

Aberrant DNA methylation during carcinogenesis has been studied for a long period in the past decades. The roles of DNA methylation during carcinogenesis are complex (Patai et al., 2012; Moore et al., 2013; Duruisseaux and Esteller, 2018). A study revealed that gene body DNA methylation systematically enhanced gene expression, as hypermethylation in promoter regions inhibited transcription factor binding and silenced specific genes (Yang et al., 2014). Intergenic DNA methylation influences spurious transcription initiation (Neri et al., 2017). Moreover, DNA methylation in nuclear territories could transform euchromatin into heterochromatin and inhibit gene activation (Bártová and Kozubek, 2006). In addition to gene suppression, some studies also revealed that promoter DNA methylation also recruited specific transcription factors and promoted gene expression (Qiu et al., 2016).

Seeking DNA methylation biomarkers for cancer diagnosis has been emphasized in the past few years (Dor and Cedar, 2018; Pan et al., 2018; Ding et al., 2019; Li et al., 2019b). However, DNA methylation is characterized by tissue heterogeneity. The methylation signatures are maintained from cell-of-origin during carcinogenesis (Zhang and Huang, 2017; Bormann et al., 2018), leading to DNA methylation transformation among cancers that are different. Since the concept that the eight hallmarks of cancer are widely accepted (Hanahan and Weinberg, 2011), it is aimed to find common DNA methylation signatures as biomarkers. Xiaofei et al. (Yang et al., 2017) found four universal hypermethylated CpG sites in DRD5 promoter regions, while only one of them (cg22620090) was differentially methylated in all 15 cancers. Saghafinia et al. (2018) identified hyper- and hypomethylated sites and found converged pathways between genetic and epigenetic alterations. Researchers also developed an algorithm using deep learning for normal-cancer discrimination using DNA methylation sites and achieved about 90% sensitivity and specificity (Liu B. et al., 2019).

However, the past studies focused on the DNA methylation sites shared across cancer types or the DNA methylation pattern of a single gene in some specific cancers (Li et al., 2013, 2019b; Ding et al., 2019). The former cannot give insight into the putative mechanism that how the shared methylation sites occur (Saghafinia et al., 2018), while the latter fails to achieve a pan-cancer signature (Li et al., 2019b). In this study, we identified differentially methylated sites (DMSs) and regions (DMRs) across cancers. Then we analyzed the downstream gene expression and the three-dimensional (3D) chromatin structure to validate the findings. We found that HOXA locus DNA hypermethylation is a commonly shared signature across cancers. The signature causes aberrant chromatin structure and thus influences the co-expression pattern of genes in the HOXA locus.

## Materials and Methods

### Data Collection

The Cancer Genome Atlas (TCGA) DNA methylation data evaluated by Illumina Human Methylation 450K BeadChip were collected in May 2020 to reduce the batch effect of different platforms. All samples and cohorts were enrolled in this step. The cohorts with a tumor or normal tissues sample size less than 30 were excluded in this step. Only primary tumors were enrolled in tumorous samples, while metastatic or recurrent tumors were omitted to reduce the redundancy with its primary tumor samples.

In Gene Expression Omnibus (GEO) datasets, the datasets were searched with keywords “cancer” or “tumor” and then filtered with Illumina Human Methylation 450K BeadChip. The “Entry type,” “Organisms,” and “Attribute name” were set as “Series,” “Homo Sapiens,” and “Tissue,” respectively, in May 2020. Subsequently, each dataset was annotated manually. The dataset with a sample size of less than 60 was excluded. The datasets that were annotated with the same cancer type but not in the same cohort were combined for further analyses.

### Data Pre-processing and Normalization

The data were downloaded in the raw data format. The normalization method was selected as quantile. Due to the large sample size, the normalization step was not performed with current R or python packages, which are not available for the file size. In-house scripts were used in this step. Since all samples originated from TCGA and GEO datasets were evaluated with Illumina Human Methylation 450K BeadChip, the probes used were identical. The average values for each probe were calculated in all samples enrolled. The average probe values were sorted and assigned to each sample according to the ranks of probes.

### Differentially Methylated Site and Region Identification

The normalized data were read, and the DMRs were identified using the R package “minfi” (Fortin et al., 2017). The adjusted p values were set as 0.01. Considering that CpG sites in the Illumina Human Methylation 450K BeadChip platform were predetermined and that the DMSs consist of a large proportion of all sites in the platform, another parameter for screening was used. To evaluate the diagnostic value of each CpG site to discriminate the cancerous and normal tissues, the area under the receiver operating characteristic (AUROC) curve (AUC) was employed for further identification. The average threshold AUCs of each probe across cancers were set as 0.75. Regions with over 10 coordinately contiguous CpG sites were identified as DMRs. Since the annotation of Illumina Human Methylation 450K BeadChip is based on hg18, the coordinates were converted to hg19 using the UCSC tool “liftover.”

### Gene Expression and Hi-C Data Processing and Visualization

Gene expression data of TCGA cohorts were downloaded from UCSC Xena (Goldman et al., 2020)^1^ website. Gene expression was estimated as log2(x + 1) transformed RSEM normalized count. The expression values of HOXA genes (HOXA1, HOXA2, HOXA3, HOXA4, HOXA5, HOXA6, HOXA7, HOXA9, HOXA10, HOXA11, and HOXA13) were retrieved. The gene expression correlations of the genes were calculated using the “Pearson” correlation method on the R platform, and the result was visualized with heatmap plotted by R package “pheatmap.” The correlation matrix was plotted for all 11 cancer types.

Hi-C data were retrieved and visualized using “juicebox” (Robinson et al., 2018), which collected the Hi-C data of cell lines and tissues in September 2020. Among these datasets, only human cell lines mapped with the hg19 genome version were used for further analyses. If samples have replicates, the replicates were combined. The target region for display was selected as upstream 200 kb of DMRs to its downstream 200 kb (DMRs ± 200 kb). The normalization method for each sample was used as “Balanced.”

### Statistical Analyses and Software

All analyses were performed with R language^2^ (version 4.0.2, 2020-6-20). The AUCs were calculated using the R package “pROC” (Robin et al., 2011). Survival analyses were implemented using package “survival.” The correlation matrix was plotted with package “pheatmap.” The Manhattan plot was drawn using the R package “qqman.” The Hi-C data were downloaded and visualized with software named “juicebox,” and relative regions of HOXA genes were visualized with UCSC genome browser (Kent et al., 2002). During analyses, *p* < 0.05 was considered to be statistically significant.

## Results

### Data Collection

All DNA methylation datasets evaluated by Illumina Human Methylation 450K BeadChip in TCGA cohorts were used in this study. Datasets with a sample size of 30 or more per group (normal and tumor groups) were retained. As a result, 10 cancer types, including breast cancer, colorectal cancer, head and neck carcinoma, kidney clear cell carcinoma, kidney papillary cell carcinoma, liver cancer, lung adenocarcinoma, lung squamous cell carcinoma, prostate cancer, and thyroid cancer cohorts, were enrolled in this study. Only primary tumor and adjacent normal tissues were used, while metastatic and recurrent tissues were excluded. In addition to the TCGA cohort, cancerous datasets from GEO were also collected using keyword searching and manual annotation. The detailed sample size is shown in Table 1.

**TABLE 1:** Datasets and corresponding sample number in the Cancer Genome Atlas (TCGA) and Gene Expression Omnibus (GEO) enrolled.

	Normal	Tumor	GEO accession number
GEO-Leukemia	23	63	GSE78963, GSE76585, and GSE45459
GEAO-Glioma	221	730	GSE44684, GSE73747, GSE54415, GSE45353, GSE92577, GSE120650, GSE73515, GSE114534, GSE103659, GSE79064, GSE40360, GSE78218, GSE57360, GSE90871, GSE101658, GSE49393, and GSE116754
GEO-Breast cancer	222	389	GSE108213, GSE88883, GSE72308, GSE78758, GSE74214, and GSE100653
GEO-Cervix cancer	38	7	GSE46306
GEO-Colon cancer	114	270	GSE101764, GSE68060
GEO-Esophageal cancer	73	136	GSE72872, GSE74693, and GSE52826
GEO-Head and neck cancer	59	69	GSE98807, GSE38268, GSE38266, GSE67097, and GSE109446
GEO-Prostate cancer	17	336	GSE112047, GSE47915, GSE76938, GSE83917, and GSE112047
GEO-Melanoma	497	130	GSE90124, GSE120878, GSE44661, GSE63315, GSE67097, and GSE115797
TCGA-BRCA	99	786	
TCGA-COAD	39	298	
TCGA-HNSC	51	529	
TCGA-KIRC	161	320	
TCGA-KIRP	46	276	
TCGA-LIHC	51	378	
TCGA-LUAD	33	458	
TCGA-LUSC	44	372	
TCGA-PRAD	51	499	
TCGA-THCA	57	508	

*The TCGA data are downloaded from UCSC Xena.*

### Differentially Methylated Site and Region Identification

**FIGURE 1:**
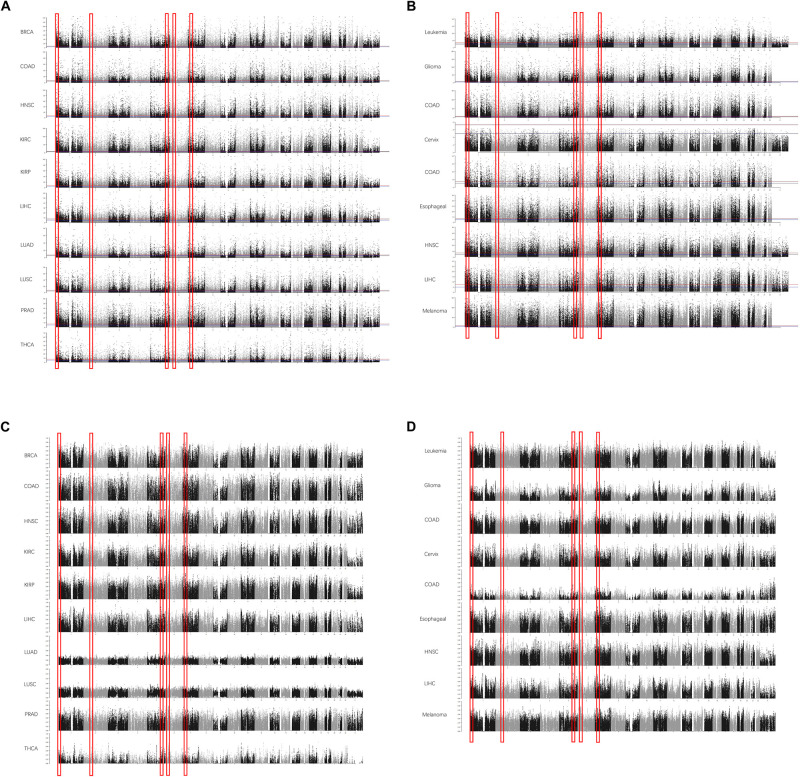
The differentially methylated sites (DMSs) and regions (DMRs) identified in cancers in Manhattan plot. Some DMSs are identified according to the false discovery rate (FDR) in The Cancer Genome Atlas (TCGA) dataset **(A)** and Gene Expression Omnibus (GEO) dataset **(B)**. The area under the receiver operating characteristic (AUROC) curves (AUCs) are also consistent with this [**(C)** AUCs in TCGA; **(D)** AUCs in GEO]. The x-axis is the coordinates in the chromosomes, and the y-axis is the FDR values **(A,B)** and AUROCs **(C,D)**.

To reduce the false-positive rate, the normal or cancerous sample size of each cancer type was set to at least 30. The beta values of each CpG were calculated. In each cancer type, the methylation level of each site was compared between normal and cancerous tissues. The DMSs and DMRs were identified. As shown in Figure 1A, some DMRs were commonly shared by different cancer types in TCGA cohorts. The regions could also be detected in the GEO validation cohort (Figure 1B). In addition to the methylation difference, the diagnostic value of each CpG site’s beta value to discriminate the normal and cancerous tissues was evaluated by the AUC (Figures 1C,D). Consistently, these regions were proved to be good discriminators using AUROCs. Detailed information for some regions is shown in Supplementary Figure 1.

Afterward, the DMRs were also identified according to the AUROCs and DMSs determined by the false discovery rate. The DMRs were defined as at least 10 coordinately continuous DMSs, whose AUROCs on discriminating the normal and cancerous tissues were at least 0.75. The candidate regions should also contain at least three CpG sites per kilobase. Accordingly, nine DMRs were identified (Table 2).

**TABLE 2:** Differentially methylated regions (DMRs) identified as common DMRs across cancers.

chrs	DMS	Density	Coordinate Hg19	Length
	number	(DMS/kb)		
chr1	12	0.01875	chr1:25130211-25130851	640
chr2	13	0.00386	chr2:53940358-53943729	3371
chr5	10	0.00388	chr5:140777418-140779995	2577
chr5	10	0.00965	chr5:140733599-140734635	1036
chr6	15	0.02717	chr6:31804202-31804754	552
chr6	19	0.03442	chr6:29629141-29629693	552
chr6	21	0.03443	chr6:30202939-30203549	610
chr7	13	0.01207	Chr7:27150841-27151918	1077
chr7	10	0.04717	Chr7:27150584-27150796	212

*The detailed information of DMRs identified, including the number of DMSs, hg19 coordinate, length, and differentially methylated site (DMS) density.*

### DNA Hypermethylation at Differentially Methylated Regions

**FIGURE 2:**
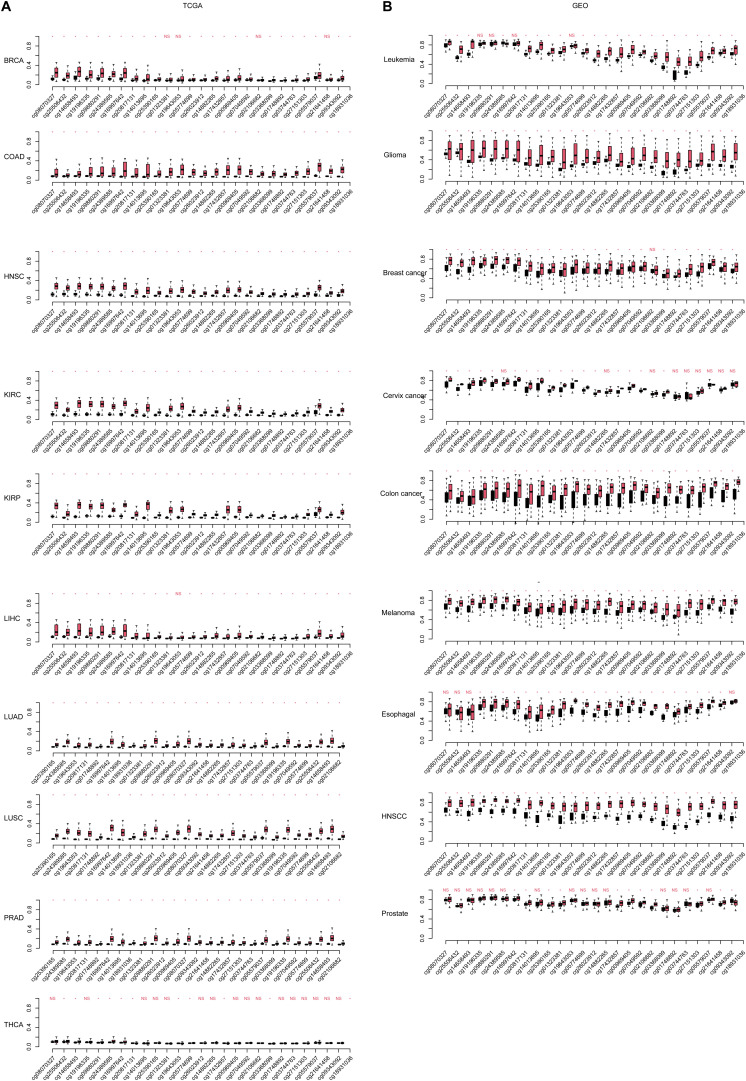
The continuous differentially methylated sites in the HOXA locus across cancers. The significance of each site in each cancer [including **(A)** The Cancer Genome Atlas (TCGA) and **(B)** Gene Expression Omnibus (GEO)] is listed. The a-axis is the CpG sites in normal and cancer (black, normal; red, cancer), and the y-axis is the relative beta-values. The CpG sites were sorted according the coordinate. “NS” indicates not significant, and “*” is statistically significant.

Among the DMRs identified, we noticed that two regions in chromosome 7 were coordinately close. These two regions spanning 1,334 kb, containing 23 DMSs, formed the highest DMS density region (CpG/kb). In the following steps, these regions were combined for further analyses. The methylation levels of CpG sites were compared between normal and cancerous tissues across cancer types. As shown in Figure 2A, CpG sites in this combined region were significantly differentially methylated in TCGA cohorts. These results could also be consistently observed in the GEO cohort (Figure 2B), despite that some cancer types were not overlapping. Most CpG sites in this region were hypermethylated in TCGA cancer cohorts, compared with the normal cohorts. This finding is reproductive in GEO cohorts. These results collectively indicate that aberrant DNA methylation in the selected region is a common signature during carcinogenesis.

### Genes Inside HOXA Locus Have Dysregulated Co-expression Pattern

**FIGURE 3:**
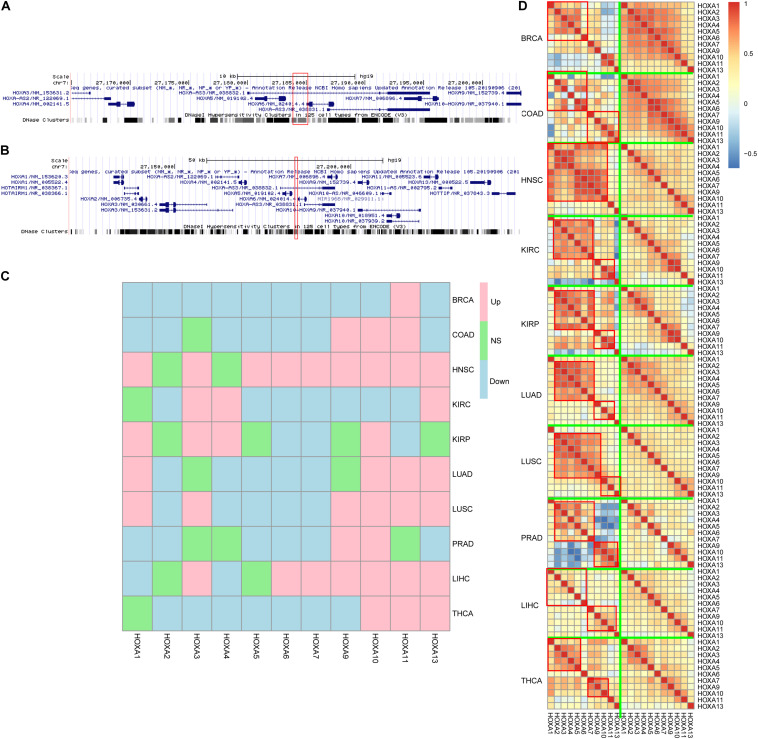
HOXA locus and gene expression. The differentially methylated regions (DMRs) located in the HOXA locus **(A,B)**, between HOXA5 and HOXA6. Despite that the genes were not consistently up- or down-regulated across cancers **(C)**, the co-regulation pattern decreased during carcinogenesis **(D)**. “Down,” “Up,” and “NS” refer to significantly down-regulated, significantly up-regulated, and not significant.

Next, the region was mapped to the human reference genome and was found to be located at the HOXA locus. Interestingly, instead of the HOXA locus boundary, this region was mapped inside the locus (Figure 3A) by separating the HOXA genes into two parts. One part contains HOXA1, HOXA2, HOXA3, HOXA4, and HOXA5, while the other part consists of HOXA7, HOXA9, HOXA10, HOXA11, and HOXA13. The three prime untranslated region (UTR) of HOXA6 is inside this region (Figure 3B).

Classically, aberrant DNA methylation is related to dysregulated gene expression. Thus, the gene expression differences between normal and cancers were compared to evaluate the impact of hypermethylation in this region. The results were somewhat controversial. On the one hand, HOXA genes were differently expressed across cancers, which was consistent with the aberrant DNA methylation profile in this region. On the other hand, the gene expression trends during carcinogenesis across cancers were not similar, while some genes were up-regulated and some were down-regulated (Figure 3C) across cancers.

An important role for DNA hypermethylation is euchromatin–heterochromatin transformation, which is reflected by gene co-expression patterns. Thus, gene co-expression patterns of the HOXA locus across cancers were analyzed. As shown in Figure 3D, HOXA gene expression was modularized in normal tissues but transformed to be stochastic in cancerous tissues. In normal tissue, HOXA2, HOXA3, HOXA4, HOXA5, and HOXA6 were strongly co-expressed, and so were HOXA9, HOXA10, HOXA11, and HOXA13. However, the co-expression pattern was disrupted in cancerous tissues. The modularized co-expression clusters were separated at HOXA6 or HOXA7, which was consistent with our finding that UTR of HOXA6 was hypermethylated. Thus, we suspected that DNA hypermethylation of this region disrupted the modularized co-expression pattern in cancers.

### 3D Chromatin Structure of HOXA Locus During Carcinogenesis

**FIGURE 4:**
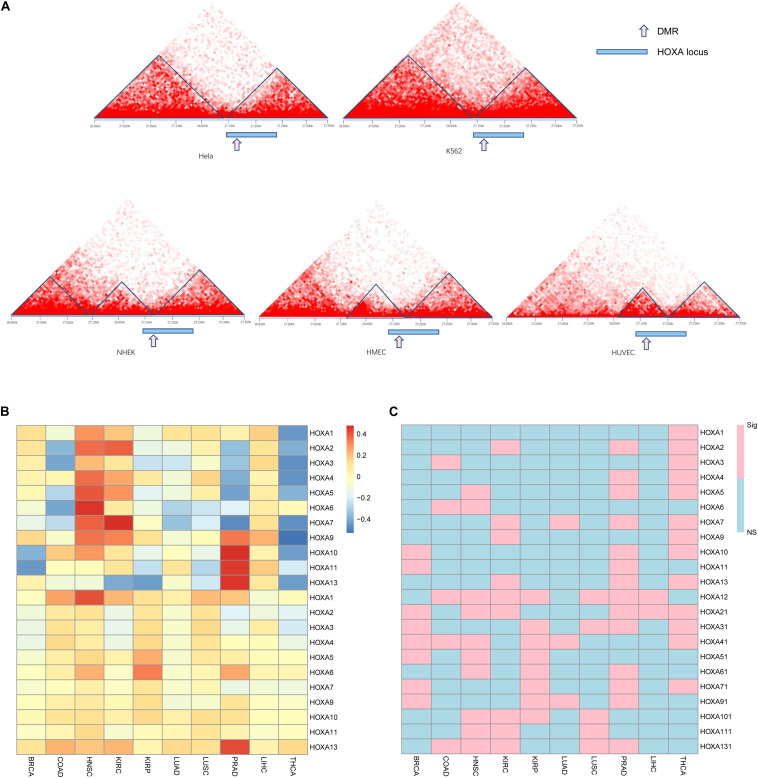
Gene regulation pattern in cancerous and normal cell lines. The topological regions **(A)**, relative sites of differentially methylated regions (DMRs), and the HOXA locus was shown. The upper panel is cancer cell lines, and the lower panel is normal cell lines. The Pearson correlation between TAX1BP1 **(B)** and *p* values **(C)** is shown. “NS” indicates not significant and “Sig” statistically significant.

Observing the disrupted modularized co-expression pattern, which may be caused by hypermethylation of this region, we seek to investigate if there exists 3D DNA structure transformation in this region during carcinogenesis of cancers. Several cancer cell lines and normal cell lines from public database were used for validation. As shown in Figure 4A, downstream of the topologically associating domain (TAD) contains a part of the HOXA locus in normal tissues while the other part is not. The boundary between the parts is located at the DMR we identified. However, the boundary inside the HOXA locus cannot be detected in cancerous tissues. Since the HOXA family locus was combined with the downstream TAD, it was promoted to investigate the co-expression correlation between this gene and the downstream genes. Here, we selected the nearest gene TAX1BP1, locating about ∼700 kb away from the HOXA locus. As shown in Figures 4B,C, the expression of TAX1BP1 was significantly associated with most HOXA family genes across cancers, but not in normal tissues. Besides, the TAD upstream is also changed. These results collectively indicate that the hypermethylation at the HOXA locus affects 3D chromatin structure and regulates gene expression.

## Discussion

DNA alterations are considered to be a driving force for carcinogenesis. The correlation between genetic alteration and 3D nuclear structure has been investigated. The spatially proximate somatic co-mutations tend to affect cancer driver genes and benefit cancer cells in growth and metastasis (Shi et al., 2016). Further classifying somatic mutations into active and inactive domains help discover the interplay between 3D genome organization and carcinogenesis (Akdemir et al., 2020). In addition to genetic alterations, epigenetic alterations are also reported to re-configure nuclear territories (Lee et al., 2019; McLaughlin et al., 2019). This study aims to find commonly altered methylation regions during carcinogenesis.

HOXA family has been widely reported for their prognostic role in tumorigenesis. For example, HOXA1 gene expression level is reported to enhance proliferation and metastasis in prostate cancer (Wang et al., 2015), breast cancer (Liu J. et al., 2019), and gastric cancer (Yuan C. et al., 2016) and predict poor prognosis in non-small-cell carcinoma (Zhang Y. et al., 2018). Abnormal DNA methylation of HOXA2 promoter regions causes dysregulation of gene expression, therefore influencing the invasion, prognosis, and clinical characteristics of colorectal cancer, glioma, and nasopharyngeal carcinoma (Li et al., 2013, 2019a; Liu et al., 2020). The reports regarding HOXA3 are controversial across cancers. It is shown to promote tumor growth in colon cancer (Zhang X. et al., 2018). However, it is down-regulated in lung cancer during carcinogenesis (Gan et al., 2018). In the meantime, abnormal promoter DNA methylation was also reported. Similarly, the role of HOXA4 gene expression during carcinogenesis, prognosis, and drug resistance is also different among cancers (Bhatlekar et al., 2018; Miller et al., 2018; Tang et al., 2019). The gene expressions and functions of HOXA5 (Ordóñez-Morán et al., 2015; Zhang et al., 2017; Peng et al., 2018), HOXA6 (Wu et al., 2018, 2019), HOXA7 (Tang et al., 2016), HOXA9 (Fu et al., 2017; de Bock et al., 2018), HOXA10 (Chen et al., 2019; Hatanaka et al., 2019), and HOXA11 (Zhang R. et al., 2018) are also controversial across cancers, except for HOXA13 (He et al., 2017). Specifically, 3′ non-coding HOXA mutation was detected in acute myeloid leukemia (Akdemir et al., 2020). In this work, our results revealed that the inconsistency resulted from a universal 3D chromatin transformation in the HOXA locus that may be caused by hypermethylation in this region.

The epigenetic alteration may be used as a biomarker for cancer diagnosis. In recent years, epigenetics, especially DNA methylation, has been emphasized (Tsou et al., 2002; Hao et al., 2017). Due to the tissue-specific pattern, DNA methylation was used to discriminate the tissue of origin for cancers (Yuan Y. et al., 2016; Kang et al., 2017), along with genomic signatures (Yuan et al., 2018). However, cancer alert is an essential step for an accurate diagnosis. Thus, the pan-cancer biomarker is still a crucial issue for cancer diagnosis. Our study may be used for cancer alert if the ctDNA of HOXA regions can be detected in the plasma.

One of the limitations of this study is that the direct causality between DNA methylation and chromatin structure cannot be validated due to current technology limitations. Another limitation is that despite global DNA methylation normalization among batches, the batch effect caused by operations during experiments, especially in the same cancer type derived from the GEO database, in which a cohort may consist of several datasets, cannot be removed.

## Data Availability Statement

The original contributions presented in the study are included in the article/supplementary material, further inquiries can be directed to the corresponding author/s.

## Author Contributions

LL, GL, and ZL designed the study. GL, YL, and XX analyzed the data. GL, XS, and LL interpreted the results. GL and XX visualized the results. All authors wrote and revised the manuscript.

## Conflict of Interest

The authors declare that the research was conducted in the absence of any commercial or financial relationships that could be construed as a potential conflict of interest.

## Footnotes

1 https://xenabrowser.net/datapages/?hub=https://tcga.xenahubs.net:443

2 www.r-project.org
