# Supplementary figures and images for: Pan-Cancer Genome-Wide DNA Methylation Analyses Revealed That Hypermethylation Influences 3D Architecture and Gene Expression Dysregulation in HOXA Locus During Carcinogenesis of Cancers
Source: Front Cell Dev Biol. 2021 Mar 18;9:649168. doi: 10.3389/fcell.2021.649168 (PMC8012915; doi:10.3389/fcell.2021.649168)

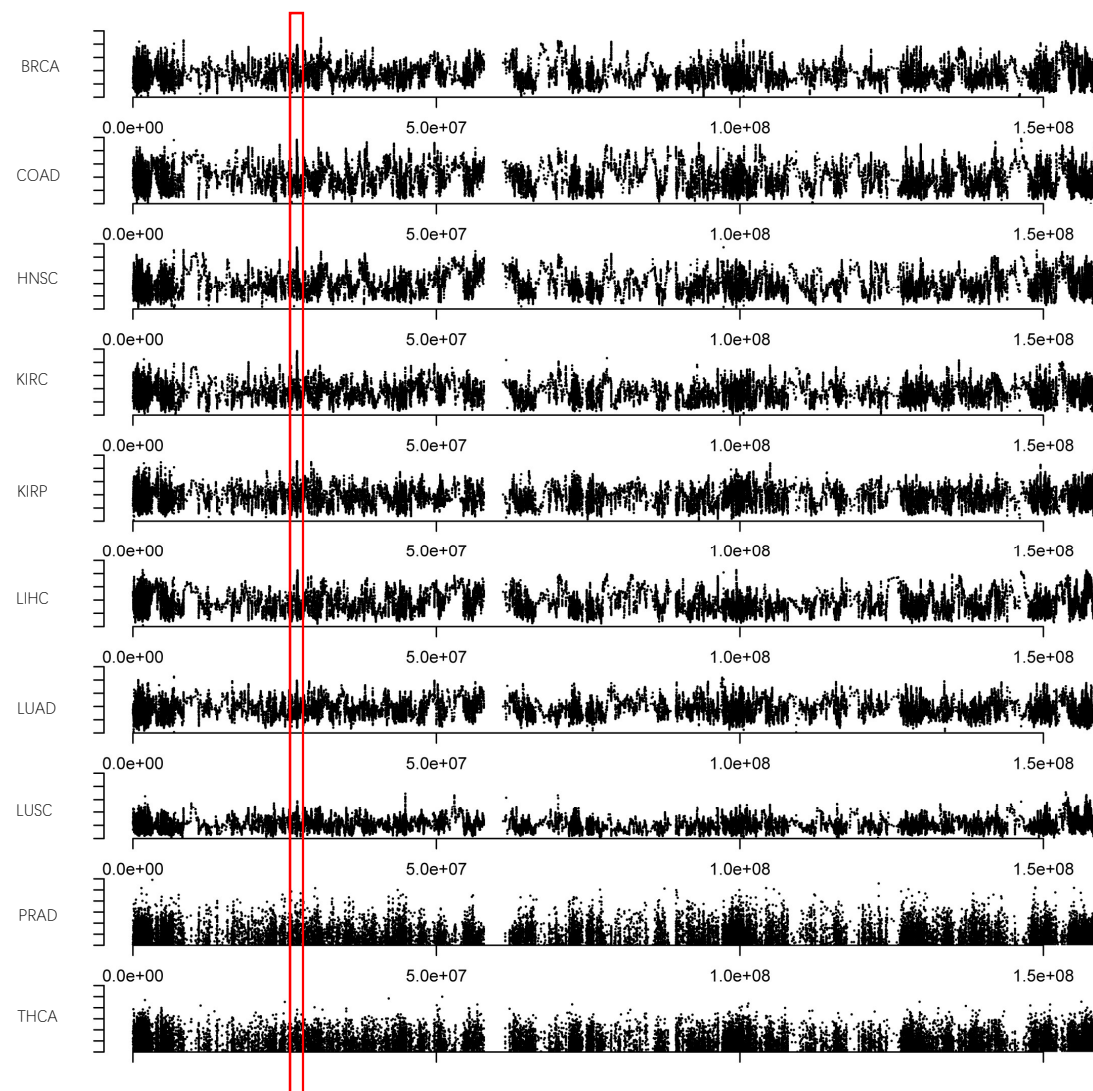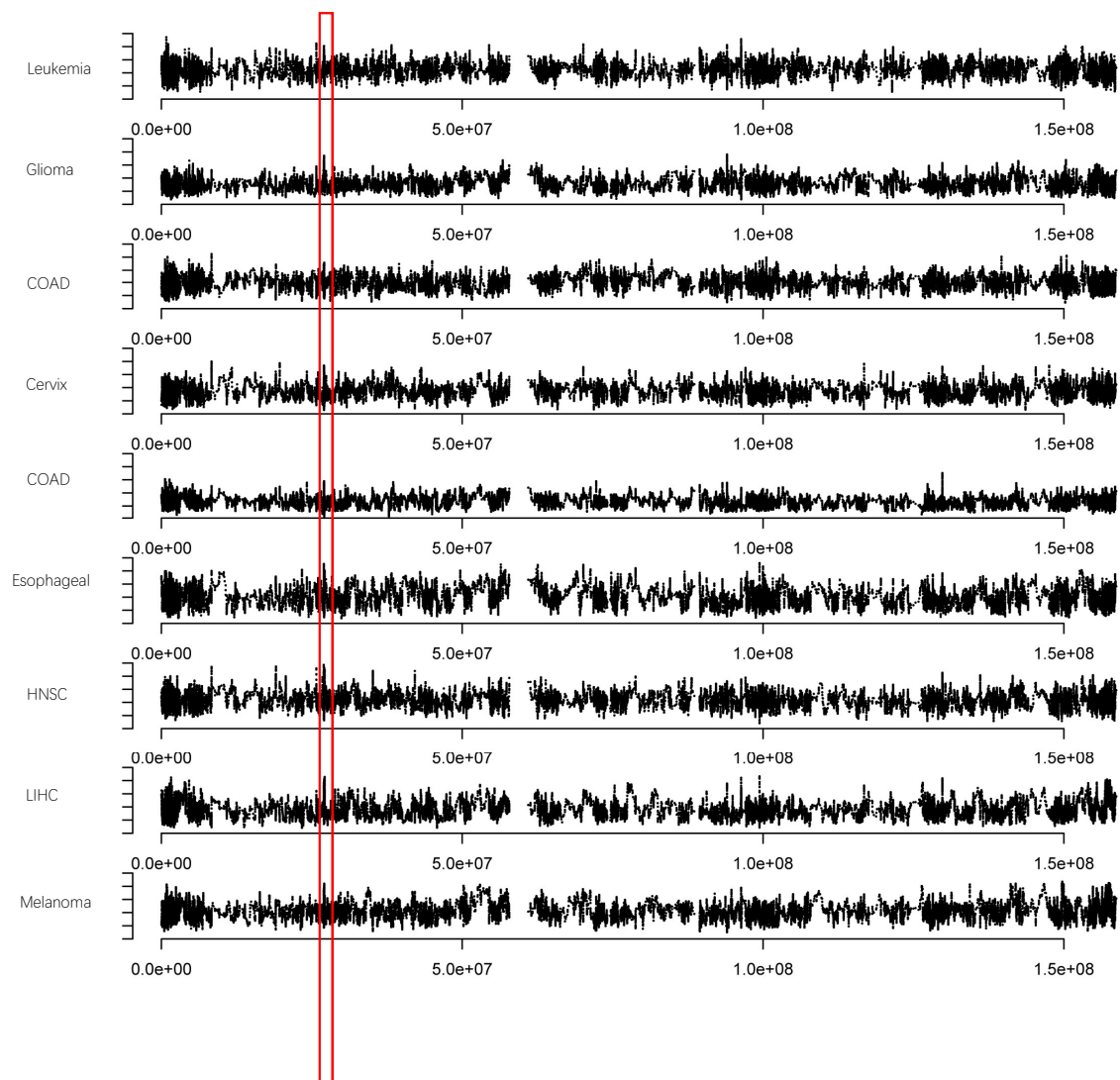

Supplement: Supplementary file 1 [file Image_1.pdf]
